# Utilising Virtual Clinics and Orthoptists to Aid COVID-19 Service Recovery in Adult Strabismus

**DOI:** 10.22599/bioj.273

**Published:** 2022-11-07

**Authors:** Jessica E. Francis, Martin Rhodes, Joshua Simmons, Jessy Choi

**Affiliations:** 1 Royal Hallamshire Hospital, GB

**Keywords:** Telemedicine, Strabismus, Orthoptist: AHPs, COVID-19, Sustainability

## Abstract

**Background:**

The Sheffield Virtual Adult Strabismus service was already well established and was put to real-time trial during the COVID-19 pandemic. We describe a multi-disciplinary adaptation to offer a safe and effective service delivery. We evaluate the efficacy of a virtual strabismus service during the pandemic to meet clinical demand, streamline patient care, balance care delivery and optimise medical input.

**Methods:**

Prospective data analysis from the virtual strabismus clinics dated from January 2015 to November 2021. All information was captured at first consultation with comprehensive specialist Orthoptic assessment and imaging; then reviewed by a strabismus consultant for clinical outcome. Management was discussed virtually with patients by the consultant.

**Results:**

Pre-COVID (January 2015–March 2020), 1,068 appointments were offered. During COVID (July 2020–November 2021), 442 appointments were offered. Clinical capacity increased to meet demand. Within two months of service re-opening, first appointment mean waiting time reduced below 18 weeks. During COVID, 24.6% of patients were listed for procedures after first visit. Face-to-face medical follow up for non-surgical cases reduced from 47.7% to 16.3%.

**Conclusion:**

Virtual strabismus services offer flexible, safe and effective ways to meet fluctuating referral patterns and maximise limited time and resources. Orthoptists are uniquely essential and highly valued keyworkers to conservatively manage non-surgical strabismus. Utilising the skillsets of Allied Health Professionals (AHPs) across the NHS is crucial to sustain ongoing clinical demand and patient care.

## Introduction

Ophthalmic specialities such as medical retina and glaucoma services have long successfully adopted virtual clinic settings into standard practice (Kortuem et al., 2017; Kotecha et al., 2017). During the COVID-19 pandemic, multiple NHS specialities quickly embraced virtual clinics as a safer alternative to traditional face-to-face clinical assessment (Kilduff et al., 2020; Puttasiddaiah et al., 2021). Virtual clinics also offer an ideal solution to cope with the backlog generated during the COVID-19 pandemic and the impacted workforce.

The Adult Strabismus service at Sheffield Teaching Hospitals (STH) has utilised virtual clinics since 2015 (Choi & Rossiter, 2016). These began with the aim to reduce the waiting times for new referrals whilst continuing to maintain an optimum standard of care. During the COVID-19 pandemic, this pathway evolved further to reduce patient and staff exposure, sustain high quality patient management and streamline the surgical listing and discharge processes.

Orthoptists are AHPs and autonomous practitioners. The core role of an orthoptist involves diagnosing, investigating and managing patients with eye movement disorders and visual development problems. The role of the orthoptist significantly expanded within the last decade to support the growing clinical pressures within ophthalmology. Many orthoptists work within advanced clinical roles in specialities such as: medical retina, glaucoma and neuro-ophthalmology. The skillset of an orthoptist has developed to include: the delivery of intravitreal injections and intramuscular botulinum toxin injections, YAG laser capsulotomy and practise as first assistant in strabismus surgery, amongst other skills.

## Virtual Care Pathway in Sheffield Adult Strabismus

**Figure 1:**
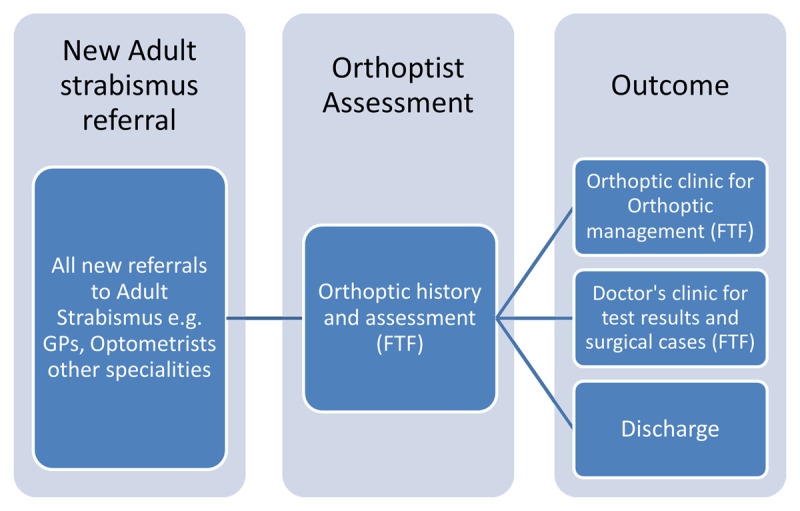
Adult strabismus care pathway before the COVID-19 pandemic, TC = telephone consultation, FTF = face-to-face.

Most patients seen in the Sheffield Adult Strabismus service were referred directly from community optometrists (High-street Optician) and general practitioners (GPs). All new referrals were triaged and graded by a team of designated senior orthoptists. The first clinical appointment of the virtual service consisted of a face-to-face bespoke Orthoptic assessment and ophthalmic imaging. Orthoptic assessment included: visual acuity, prism cover test measurements in five positions of gaze, ocular motility and binocularity testing, pupil check and intraocular pressure measurement. Ophthalmic imaging included: wide angle retinal imaging and photos in nine positions of gaze. Review of the notes was completed by the strabismus consultant. The patient was then contacted by the consultant by telephone to discuss the management of their individual case in depth or to discharge if appropriate. The clinical summary letter was sent to the patient and the GP to outline appropriate management and follow up plan if indicated (Figure 1).

## COVID-19 Changes to Fast Track Care Pathway

**Figure 2:**
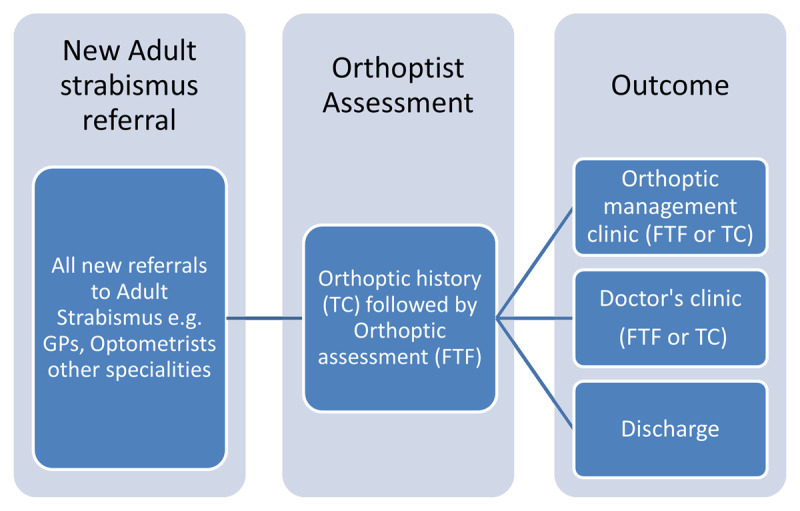
Adapted adult strabismus care pathway during COVID-19 Pandemic, TC = telephone consultation, FTF = face-to-face.

Changes were made to facilitate the safe reopening of routine clinical practice, comply with hospital trust social distancing policy, and cope with grossly reduced staffing levels due to COVID related illness, home working for the clinically vulnerable and staff redeployment. The new model was trialled when the department reopened to non-urgent, routine clinical referrals (Figure 2). The changes implemented included:

- Surgical listing for corrective eye muscle surgery, intramuscular botulinum toxin injection and bupivacaine injection was undertaken by the consultant during virtual clinic telephone review after the first clinical orthoptic assessment. Patients then received a squint surgery patient information leaflet and letter to confirm their surgical listing via post. This revised the pre-pandemic historical practice of at least three face-to-face eye movement assessments prior to surgical listing, and thus reduced the number of hospital visits required. Patients attended again for a pre-operative orthoptic assessment, this included prism cover test measurements in five positions of gaze to establish deviation stability, prior to corrective eye muscle surgery. The majority were performed with adjustable suture technique.
- Orthoptists took patient histories with a standardised template during a telephone triage appointment prior to clinical assessment. This significantly reduced the face-to-face contact time during examination and the associated risk of prolonged exposure at the peak of the pandemic.
- Clinic capacity was maximised by booking orthoptic assessments into any available slot throughout the week; previously, assessments were only scheduled alongside consultant strabismus clinics. The capacity was limited by clinic space and staffing levels. The virtual care pathway had an inbuilt failsafe default for any patients of acute clinical concern. These were assessed by the emergency eye centre and the on-call ophthalmologist to assess the need to escalate immediately. Examples included patient presentation with acute painful 3^rd^ cranial nerve palsy, acute dysthyroid eye disease, bilateral 6^th^ cranial nerve palsy with papilloedema or features indicative of giant cell arteritis.
- Orthoptic follow-up telephone clinics were introduced to manage patient education in convergence insufficiency and to assess patient’s response to prism management. This negated the need for unnecessary appointments and allowed prioritisation of patients requiring assessment.

## Materials and Methods

Prospective data was kept since the start of the virtual adult strabismus service. Analysis of all referrals between January 2015 and November 2021 was undertaken. January 2015 to March 2020 was considered ‘pre-COVID’ while July 2020 to November 2021 was considered ‘COVID’. Consultant led strabismus clinics ceased between April 2020 and June 2020, as resources were re-distributed and allocated elsewhere to tackle the increasing demands of the COVID-19 pandemic.

A total of 1,068 appointments were offered on the virtual strabismus pathway during January 2015–March 2020 (pre-COVID), 974/1,068 appointments were attended, and 962/974 (98.8%) of records were captured in the database. During July 2020–November 2021 (COVID) 309 appointments were offered, 293/309 appointments were attended, and 252/293 (86.0%) of records were captured in the database. The missing data was attributed to the workforce rotation, leading to some data not being captured in the database. An additional stream of virtual clinics was set up for a second consultant from January 2021 to distribute the increasing workload. The total number of appointments offered on combined consultant stream during COVID was 442, 418/442 appointments were attended.

All records captured in the database were from the first consultant stream. Records in the database were analysed for: waiting time for first appointment, appointment outcome, patient diagnosis and referral source. Assessment of impact was measured by the waiting time for appointment (in weeks) and percentage reduction of consultant face-to-face clinical consultations. This service evaluation was registered with the Clinical Effectiveness Unit on 7th June 2021. It was given a Sheffield Teaching Hospitals project number: 10615.

## Results

**Figure 3:**
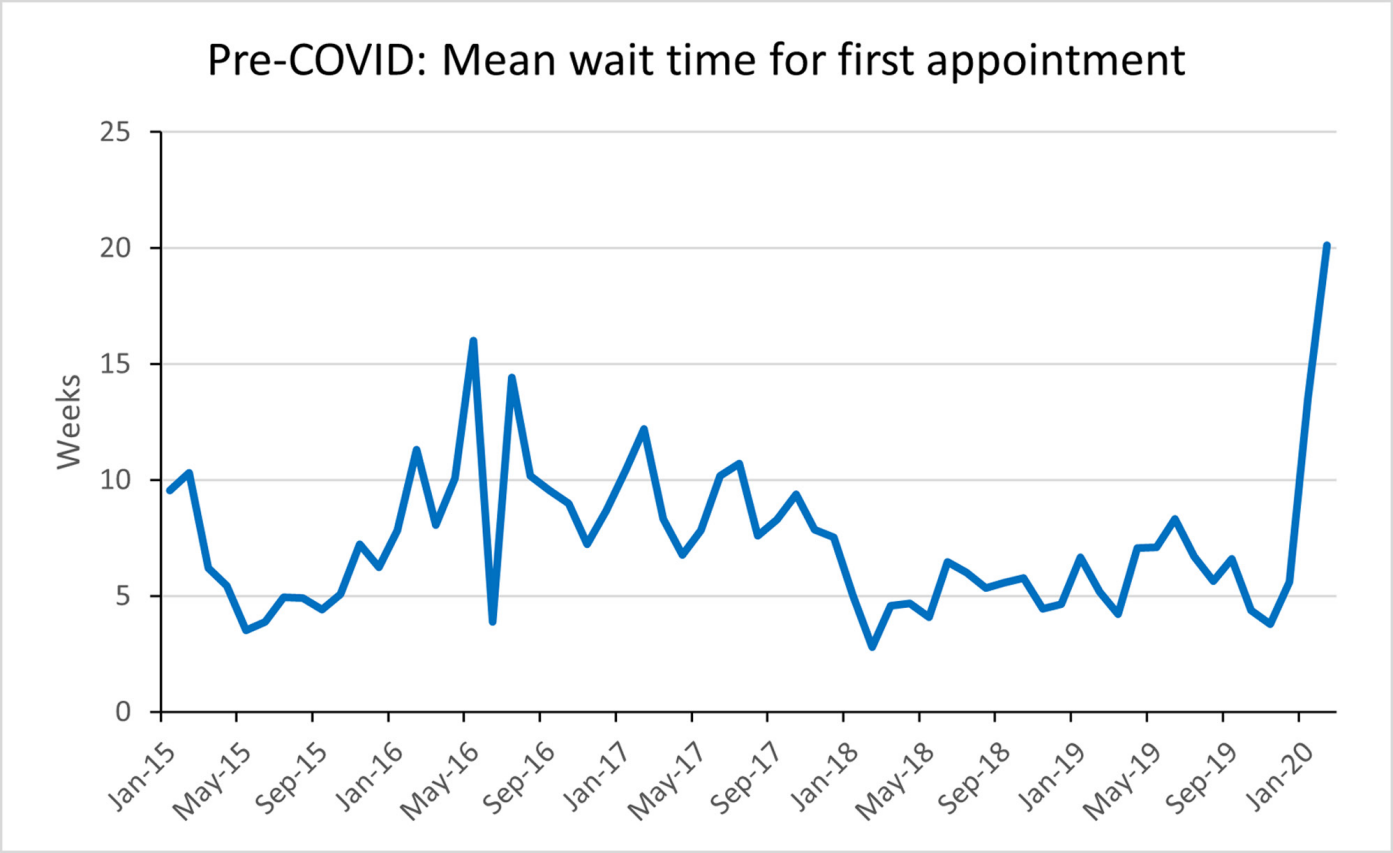
Mean wait time for first appointment for new referrals into the adult strabismus service between January 2015–March 2020.

**Figure 4:**
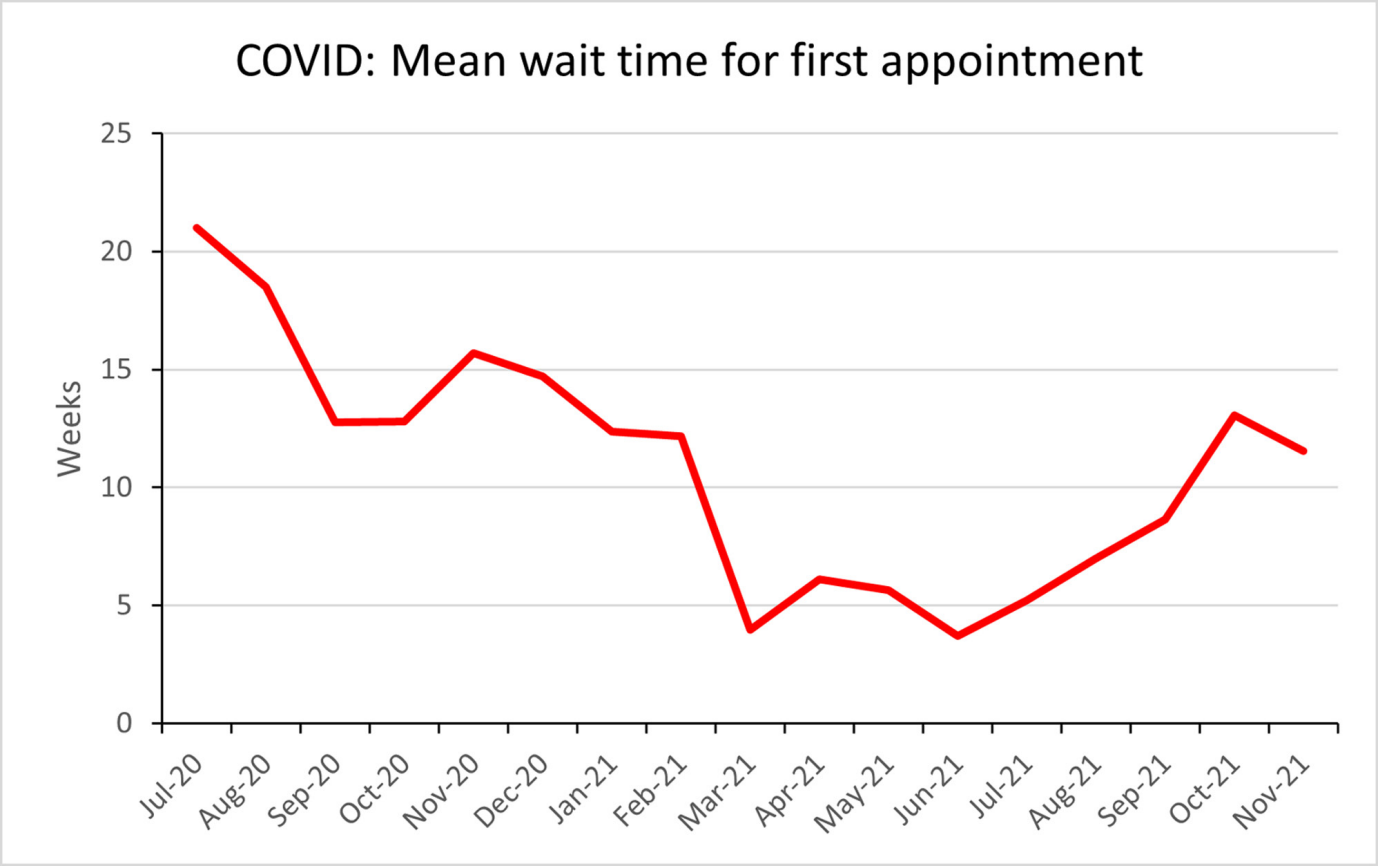
Mean wait time for first appointment for new referrals into the adult strabismus service between July 2020–November 2021.

The overall mean wait time for first appointment during the ‘pre-COVID’ period was 7.4 weeks and remained below 17 weeks until February 2020 (Figure 3). When the service re-opened in July 2020 the mean wait time was 21 weeks, this decreased rapidly to 4 weeks by March 2021. The mean waiting time during ‘COVID’ was 10.9 weeks and remained consistently below 18 weeks from September 2020. (Figure 4).

**Figure 5:**
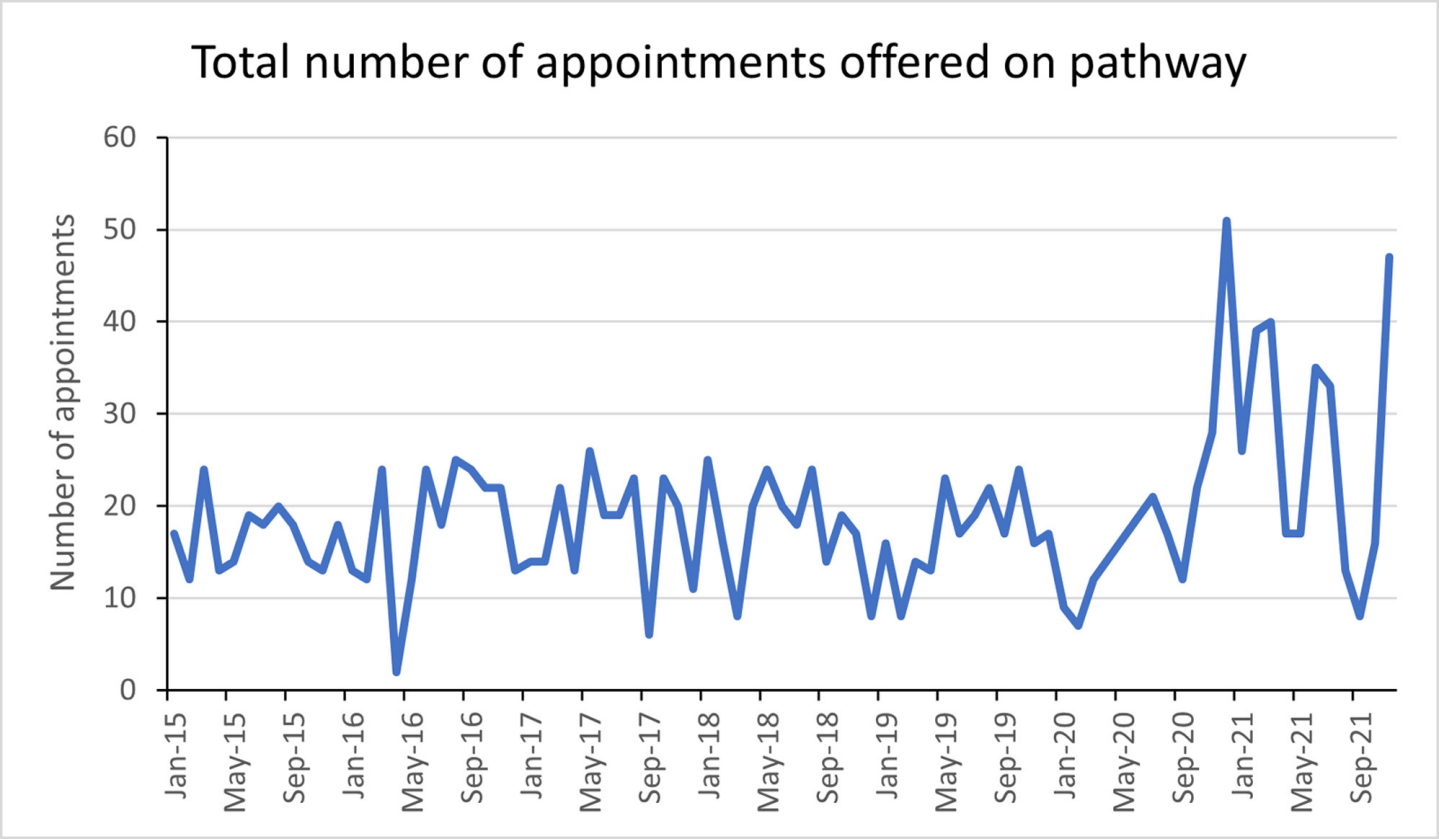
Number of appointments offered on the adult strabismus pathway between January 2015–November 2021.

Figure 5 demonstrates the ability and resilience of the care pathway to cope with dramatic changes in appointment demand. Easing of lockdown restrictions in July 2020 and June 2021 corresponded with increased attendance for routine eye examinations and a large spike in referrals. To manage appointment demand, the number of patients seen in the strabismus clinic increased accordingly. To achieve this, every available clinical slot was utilised, including ‘unpopular’ appointment times such as Friday afternoon, to make full use of clinician time. In addition to this, the temporary drawing back of certain services allowed a great deal of diversity and resilience. For example, low vision clinics were scaled back due to the high proportion of clinically vulnerable patients who generally access the service.

**Figure 6:**
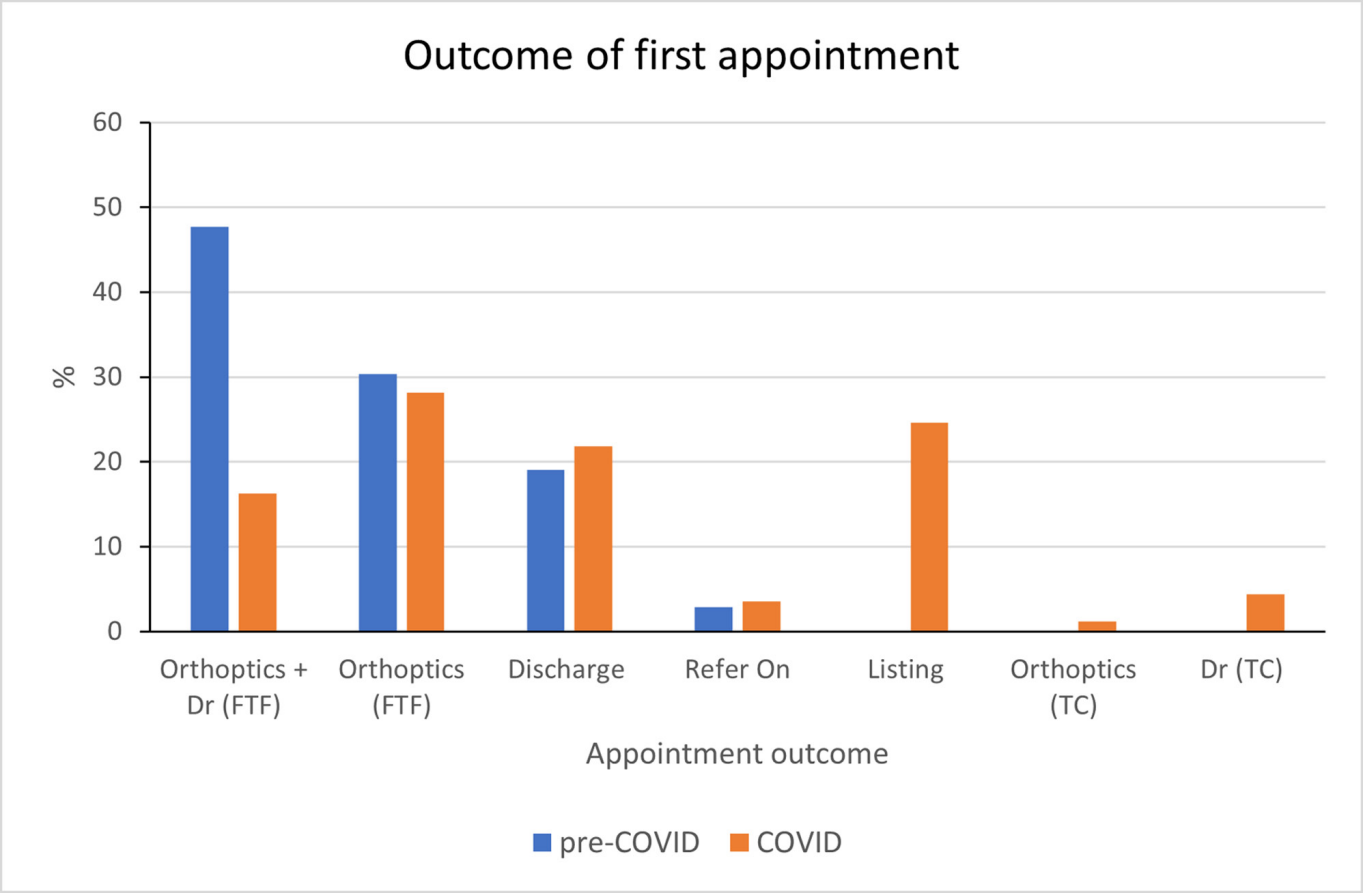
Outcome of first appointment comparison for pre-COVID and COVID periods, FTF = face-to-face, TC = telephone call.

The surgical pathway was streamlined further during COVID-19. Consequently, 24.6% of patients were listed for surgery, botulinum toxin and bupivacaine injection via telephone call with the consultant after their first face-to-face orthoptic visit (Figure 6). Prior to pathway changes, surgical listing occurred following approximately three face-to-face clinical visits. The consultant could recall one occasion when a patient was listed for a surgical procedure on their first visit prior to pathway changes, however this episode was not captured in the database.

The rate of face-to-face consultant follow up for non-surgical cases reduced from 47.7% to 16.3%. Since July 2020, 28.2% of new referrals were managed conservatively and monitored by orthoptists (Figure 6). Discharge rate after first visit increased slightly during COVID from 19.0% to 21.8%. The value of communicating the findings with the patient after the initial assessment was vital in establishing rapport and much needed reassurance that they are receiving the most appropriate care, especially in the already heightened anxiety mind-set during the pandemic. There were also more referrals for symptoms not related to eye movement defects, due to underlying reduced status of mental wellbeing.

**Figure 7:**
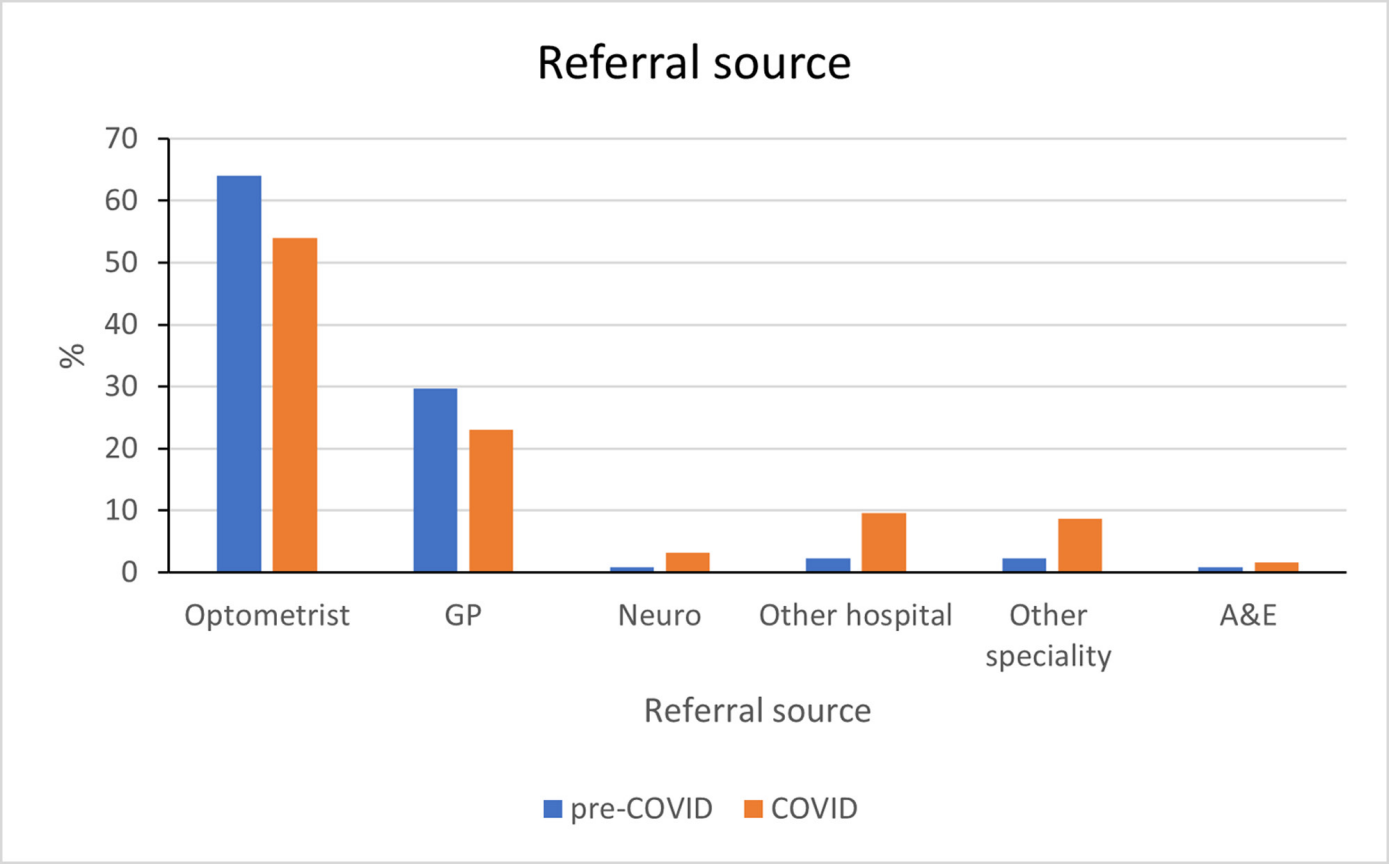
Referral source for new referrals into the adult strabismus service comparison for pre-COVID and COVID periods. Neuro = neuro-ophthalmology, GP = general practitioner, A&E = accident and emergency.

**Figure 8:**
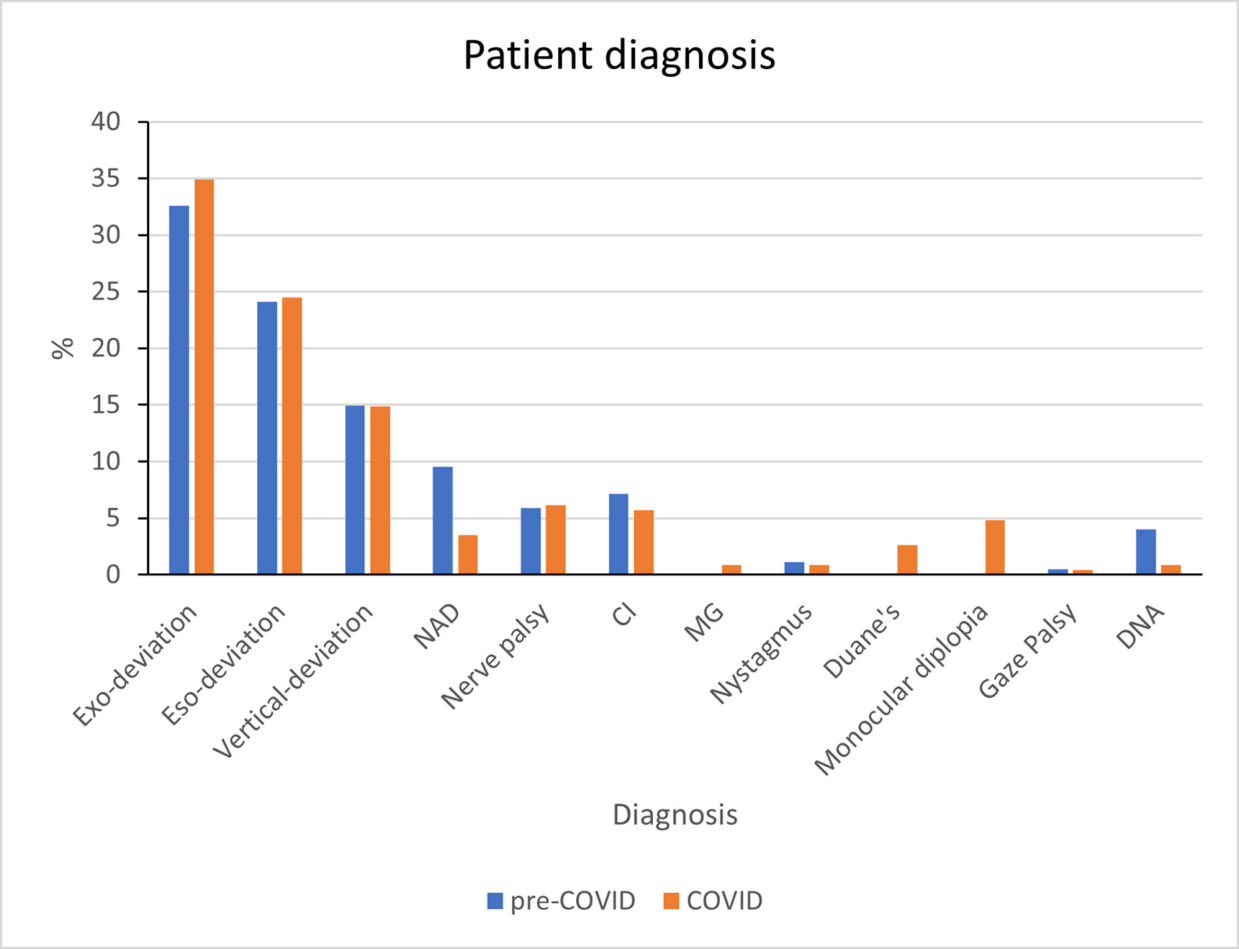
Patient diagnosis comparison for pre-COVID and COVID periods, NAD = no apparent deviation, CI = convergence Insufficiency, MG = myasthenia gravis, Duane’s = Duane retraction syndrome, DNA = did not attend.

Figure 7 demonstrates a 7.2% increase in referrals from other hospitals and other specialities during the COVID-19 pandemic, while the referrals from community Optometrists and GPs reduced, related to their overall reduced capacity during the pandemic. In addition to this, despite the increase in referrals from other centres, Figure 8 demonstrates that the nature and complexity of the clinical caseload was unaffected by COVID-19 restrictions.

Although few, certain unforeseen circumstances required versatility to overcome particular barriers. If patients were unable to be contacted (e.g., not answering the phone) they were booked in for a virtual review with a specific time slot to facilitate patient engagement at a more suitable or convenient time. If language barriers were identified, utilisation of interpreting services and family members were used to overcome this.

## Discussion

The NHS faces unparalleled challenges as hospitals attempt to return to pre-pandemic activity. All services have been disrupted by staff absence due to infection and self-isolation. Recent predications have indicated NHS waiting lists could rise to 10.8 million by December 2023 (Institute for Fiscal Studies, 2022). It is crucial to consider the need for fundamental changes to service and treatment delivery. One way this can be achieved is by utilising virtual clinics. Virtual medical review has many benefits; for example, virtual appointments are typically shorter in duration than face-to-face appointments. Consequently, more consultations can be undertaken within a clinical session, around other clinical responsibilities, and from a variety of locations to meet service needs. This maximises consultant time and clinic space.

Tertiary referral centres, such as Sheffield Teaching Hospitals, receive complex case referrals from community Optometrists, GPs and other hospitals. Strabismus patients requiring complex care are frequently referred to tertiary centres for management. It was found that during the pandemic, an increase in referrals was seen both from other hospitals, and specialties. With the burden of backlog throughout the NHS and soaring surgical waiting times in district general hospitals, the clinicians are referring less straight-forward cases more promptly and patients are seeking alternative providers with shorter waiting lists. Orthoptists are crucial in the diagnosis and management of these patients and collaborate alongside ophthalmologists and the wider multi-disciplinary team, in the presence of limited medical resources, to ensure clinical demands are met.

Surgical strabismus cases were impacted by COVID-19. Staff redeployment, social distancing measures, shortage of ventilators and limited theatre space led to a period of complete cancellation of all routine surgery, and clinical activity was reduced significantly to cope with the strain and pressure on the NHS from COVID-19 related hospitalisations. The virtual clinic pathway aims to streamline patient care and implement a COVID recovery plan.

After COVID pathway changes, 24.6% of patients were listed for procedures after their first clinical visit. A further measurement was undertaken at pre-assessment. Historical teaching advocates three measurements prior to surgery; however, this recommendation has never undergone clinical review. Many longstanding strabismus patients are stable, with two measurements likely sufficient to confirm this. Furthermore, complex patients, such as those with thyroid eye disease, frequently have previous angle measurements available from clinical assessments in thyroid consultant clinics.

This revised pathway combined a modern approach to achieve good surgical outcomes. Adjustable suture technique was utilised to achieve targeted post-operative angle, incorporating a safe and effective way to treat strabismus requiring surgical intervention. An ongoing surgical strabismus logbook was kept, this showed comparable surgical outcomes. However, an audit of the pathway and full comparison of surgical outcomes are necessary to ensure safety and surgical outcomes are maintained.

In addition, there are many patient benefits gained by streamlining clinical pathways to reduce the number of necessary appointments. For example, less disruption to education and work, and reduced travel and hospital parking expenses. Consequently, this revised pathway also promotes a more environmentally conscious and sustainable service, helping lower carbon emissions associated with hospital attendance.

The COVID pathway adaptions produced a 31.4% reduction in face-to-face doctor follow up for non-surgical cases. This released medical time to focus on eliminating the COVID follow-up backlog and on surgery. This model highlights the innovative and adaptive role of Orthoptists as key AHPs to continue delivering safe and effective patient care.

## Conclusion

NHS services face unparalleled pressure to meet the challenges presented by the profound negative impact of COVID-19. The pressure generated during the pandemic has propelled creative-mindsets into developing an alternative care-model, with long-lasting benefit to the future NHS. Virtual clinics provide a logical way forward to address the mounting waiting lists and maximise clinical time and resources. Virtual clinics have been successfully implemented within adult strabismus at Sheffield Teaching Hospitals. COVID-19 service adaptions have streamlined care for surgical cases and reduced new referral waiting time. The utilisation of orthoptists optimised consultant time and enabled greater focus on complex medical and surgical management. In conclusion, virtual clinics provide an effective way of aiding service recovery and continuing to deliver high quality, safe and prompt patient care.
